# *Shigella flexneri* Spa15 Crystal Structure Verified in Solution by Double Electron Electron Resonance

**DOI:** 10.1016/j.jmb.2010.10.053

**Published:** 2011-01-14

**Authors:** James E.D. Lillington, Janet E. Lovett, Steven Johnson, Pietro Roversi, Christiane R. Timmel, Susan M. Lea

**Affiliations:** 1 Inorganic Chemistry Laboratory, University of Oxford, South Parks Road, OX1 3QR, UK; 2 Sir William Dunn School of Pathology, University of Oxford, South Parks Road, OX1 3RE, UK

**Keywords:** nitroxide, type 3 secretion system, chaperone, spin label conformation, MMM, DEER, double electron electron resonance, MMM, multiscale modeling of macromolecular systems, MTSL, *S*-(2,2,5,5-tetramethyl-2,5-dihydro-1*H*-pyrrol-3-yl)methyl methanesulfonothioate, T3SS, type 3 secretion system, ELMO, engulfment and cell motility protein

## Abstract

*Shigella flexneri* Spa15 is a chaperone of the type 3 secretion system, which binds a number of effectors to ensure their stabilization prior to secretion. One of these effectors is IpgB1, a mimic of the human Ras-like Rho guanosine triphosphatase RhoG. In this study, Spa15 alone and in complex with IpgB1 has been studied by double electron electron resonance, an experiment that gives distance information showing the spacial separation of attached spin labels. This distance is explained by determining the crystal structure of the spin-labeled Spa15 where labels are seen to be buried in hydrophobic pockets. The double electron electron resonance experiment on the Spa15 complex with IpgB1 shows that IpgB1 does not bind Spa15 in the same way as is seen in the homologous *Salmonella* sp. chaperone:effector complex InvB:SipA.

## Graphical abstract

**Graphical Abstract:**

**Figure:**
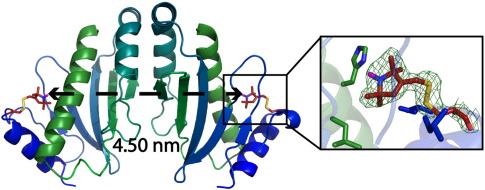

**Research Highlights:**

► A DEER measured distance between Cys19 bound spin labels in the Spa15 dimer. ► The distance was not interpretable by multiscale modeling of macromolecular systems. ► A spin-labeled crystal structure confirmed the solution DEER distance. ► The distance was unchanged upon complexation of IpgB1 effector with Spa15.

## Introduction

The type 3 secretion system (T3SS) of *Shigella flexneri* is an essential component of bacterial virulence.^1^ Effector proteins are transported in an unfolded state through a  3-nm channel^2,3^ connecting bacterial to host cytoplasm, where they act to subvert cellular processes such as cytoskeletal control and immune response for the benefit of the bacteria.^4,5^ Before transport across the T3SS, in the bacterial cytoplasm, many effectors require a chaperone to prevent any premature or incorrect interactions, to hold them in a state competent for secretion, and to aid secretion itself.^6,7^

One such chaperone of *S. flexneri* is Spa15. This belongs to a promiscuous subset of the effector chaperone class, chaperoning at least five effectors of unrelated sequence.^8,9^ One of these effectors is IpgB1, a member of a bacterial guanine exchange factor protein family defined by a conserved WxxxE motif.^10–12^ This is a bacterial mimic of RhoG, binding to the guanine exchange factor ELMO-DOCK180 complex to activate Rac1 guanosine triphosphatase, bringing about membrane ruffling of the cell, aiding the internalisation of *Shigella*.^13,14^

The structure of Spa15 was reported by van Eerde *et al.* (PDB 1RY9).^15^ It exists as a dimer of the  15-kDa Spa15 moieties, each composed of three α-helices packed against a twisted β-sheet. The small molecular weight, acidic p*I*, dimeric pairing, and structure of Spa15 are characteristic of effector chaperones, including the *Salmonella* sp. homologue InvB.^16^

Double electron electron resonance (DEER) is an increasingly used EPR experiment in the study of protein structures and protein interactions. Paramagnetic centres must be available in the protein for this technique to be used; this is often achieved through the attachment of nitroxide spin labels to cysteine residues, such as *S*-(2,2,5,5-tetramethyl-2,5-dihydro-1*H*-pyrrol-3-yl)methyl methanesulfonothioate (MTSL), developed by Hideg and coworkers.^17^ When spatially close spin-labeled cysteines (< 80 Å) are present in the structure, DEER makes use of the dipolar coupling with distance inverse cube relationship to measure their distance apart.^18,19^ Data are obtained free of dead time effects courtesy of a four-pulse sequence.^20^ Through inter-label distances, DEER can allow information such as the conformation and binding of labeled molecules to be determined.^21^ However, obtaining global structural properties of a protein from DEER first requires an understanding of the spin-label environment, so that distances may be correctly interpreted.

Insertion of MTSL into the protein will affect the conformation of the label via interactions with the surrounding protein, altering its conformation from the unbound state as viewed by Zielke *et al.*^22^ It is important that its conformation can be predicted by modeling programs used to interpret DEER measurements by predicting distances between pairs of spin labels. For this reason, research has been undertaken to crystallise spin-labeled proteins. This includes work by the Hubbell laboratory, which demonstrated protein influences on spin label conformations in T4 lysozyme crystal structures.^23,24^ Following this, it was possible to model these spin-label conformations using Monte Carlo methods and molecular dynamics.^25–27^ It is apparent, however, that many of these studies focus on orientations of a spin label contained within an α-helix. This provides a constrained environment in which to crystallise and study the label, but may lead to models that are not applicable to non-helical environments, leading to errors in their modeling. An exception to this is a crystallography and DEER study on the *Escherichia coli* translocation channel protein Wza by Hagelueken *et al.*, which showed an electron density (and DEER data) indicating a dual conformation of a non-helically placed spin label.^28^

Simulation programs have now been developed to provide structural interpretations of DEER data. The freely available program is the multiscale modeling of macromolecular systems (MMM), which predicts spin-label orientation and the resultant DEER.^29^

In this study, Spa15 has been spin labeled with MTSL, the inter-cysteine distances determined by DEER, and their interpretation sought through MMM. From comparison with PDB 1RY9, it was known that the spin-labeled cysteines in Spa15 lie in a non-helical environment providing an interesting contrast to the examples given above. At the glycerol/water glass transition temperature (175 K), MMM could not accurately predict the DEER distance experimentally obtained.

Observation of the MTSL orientation was thus achieved by determining the spin-labeled Spa15 crystal structure. This demonstrated that the distances between labels measured from X-ray crystallography and from EPR were in agreement. It also confirmed that the MMM method produced an MTSL orientation, which is not experimentally observed. An agreement between the crystallography and DEER methods confirmed the crystallographic dimer to be a relevant solution state and provides a demonstration of DEER's accuracy in distance determination. The Spa15:IpgB1 complex has also been co-purified and spin labeled. The DEER traces obtained have enabled the impact of effector binding on Spa15 to be observed. We conclude that IpgB1 binding has no global structural effect on Spa15, and that a pocket that is utilised by the homologue InvB to bind its effectors is not used by Spa15 in this complex.

## Results and Discussion

### Spa15 DEER distances were not predicted by MMM

**Fig. 1:**
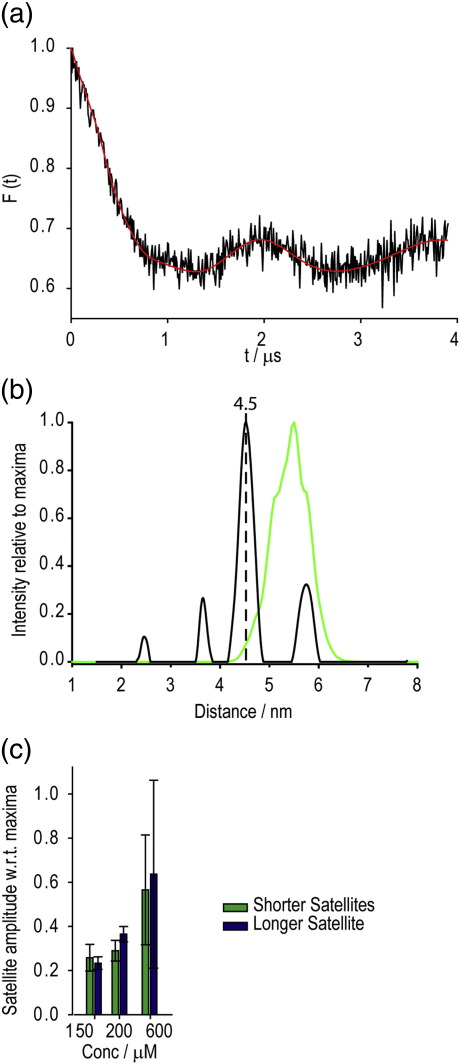
Spa15 experimental DEER data. (a) Form factor [as fitted by DeerAnalysis (red)], the dipolar evolution function after background correction for the 200 μM sample. (b) Tikhonov regularization of the form factor (regularization parameter = 1), with a primary distance of 4.5 nm seen with additional minor distance components. In green is the 5.3  nm DEER distance for the MMM most probable conformation. (c) The minor distance components changed in amplitude with the concentration of sample relative to the major 4.5  nm peak.

Spin labeling of Spa15 was achieved at 100%. This was shown by continuous-wave EPR calibration with the quantitative standard TEMPOL and verified by mass spectrometry (data not shown). DEER experiments at 50 K with 200 μM samples reproducibly identified the major distance between labels to be 4.5 nm (± 0.1 nm) (Fig. 1a and b). A fast dephasing T2 relaxation required overnight measurement despite the use of deuterated solvent. Since there was just one labeled site per monomer, the dominance of this peak indicated it as the intra-dimer label distance. A feature of the DEER spectrum was the inclusion of distances at either side of the 4.5-nm peak, which changed with sample and concentration (Fig. 1c). Other researchers have observed satellite signals to be a result of the constraining of a label, a situation identified by a variant DEER trace in an experiment upon changing the frequency difference between observer and pump frequency pulses.^30^ This possibility was ruled out, since such DEER experiments yielded identical signals between 50 and 80 MHz (data not shown). The increase in side peaks relative to the major intra-dimer peak at higher concentration led us to conclude that these side peaks were due to aggregation, a product of the preparative conditions. This was supported by multi-angle laser light scattering, which indicated that at the concentration level used in EPR, protein aggregates began to be evident (data not shown).

To try and obtain a structural understanding of this distance, we used the program MMM to predict the DEER data on the basis of the earlier structure of Spa15.^15^ MMM allows visualisation and inspection of proteins using the experimental restraints of canonical bond lengths, angles, secondary-structure elements, and structural integrity. It produces a rotamer library, calculating computationally likely conformations for MSTL in Spa15 and simulating the resultant DEER at 175 K (approximately the glass transition temperature of water/glycerol mixtures). Interestingly, the most likely conformation in this example would lead to a distance of 5.4 nm, not 4.5 nm as observed (Fig. 1b).

### Crystal structure of spin-labeled Spa15

**Fig. 2:**
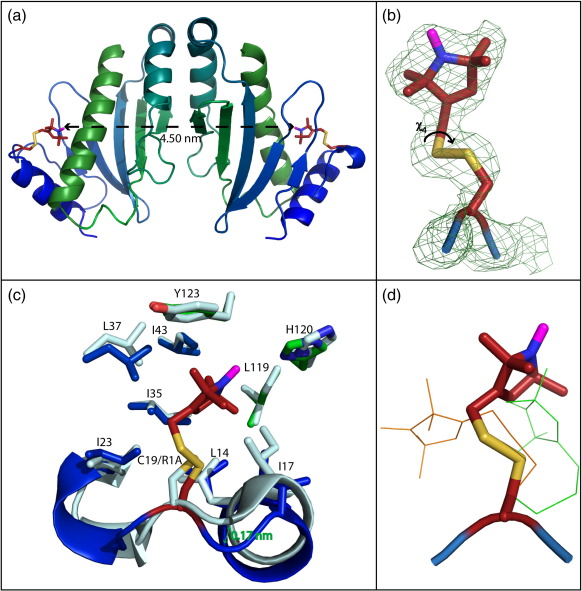
MTSL spin label observed in the Spa15 crystal structure. (a) Ribbon diagram of spin-labeled Spa15 dimer crystal structure, showing the two Spa15 subunits coloured blue to green from the N to C terminus, respectively. The MTSL spin label is atom coloured, highlighting the S–S bond, with the blue nitrogen bound to purple oxygen. The distance between the two spin labels is shown. (b) The spin label of Cys19 was highly ordered in the electron density at 1σ background. The label is shown to have only one conformation, from the MTSL O to N_D1_, due to particular stabilization by the MTSL C_6_ to C_D1_ of Ile17 and due to the steric restraints of the pocket. (c) The hydrophobic pocket within which the spin label (red) resides. Neighbouring Leu14, Ile17, Ile23, Ile35, Leu37, Ile43, Leu119, His120, and Tyr123 are shown. There is little difference between backbone structure for unlabeled (PDB 1RY9) (light blue) and labeled (colour scheme as for a) (dark blue-green) structures, with perhaps a subtle closing around the label in the latter. Side chains are similarly unaffected by the spin label, an exception being Ile17, which is placed further into the hydrophobic pocket in 1RY9 than in the labeled 2XGA structure. (d) Experimentally observed MTSL conformation (stick) compared to MMM calculated conformations. The most probable at 175 K (orange line; *P* =  0.17) is unlike the experimentally observed conformation and points away from the hydrophobic pocket. The most probable conformation at 50 K produced a DEER distance similar to that experimentally observed. However, comparison of this conformation (*P* =  0.65) with the experimental conformation shows that this is not due to the correct prediction of the experimental conformation.

The disagreement of MMM with the DEER distances caused us to turn to crystallography to explain the conformations of the spin label that provides the experimentally obtained DEER distances. Spin-labeled Spa15 was crystallised, and its structure solved (Fig. 2a).

Spa15 has three α-helices and a twisted six-stranded β−sheet, interacting with a partner via a right-angled α to α-helix hydrophobic interface, involving Leu74 and Leu78 with Ile73, Leu91, and Leu98. An extended solvent network forms at the dimer interface. Monoclinic crystals were grown and solved in the space group *P*21 using the Spa15 dimer model. The hexahistidine tag and first four N-terminal residues, as well as the loop encompassing residues 27–29, were seen to be disordered. The single spin-labeled cysteine residue at position 19 could be modeled as a single conformation with *B*-factors close to that of the main chain (Fig. 2b). The two equivalent residues are positioned at the very widest part of the heart-shaped dimer relative to one another. Due to favourable energetics, the multi-methyl substituted pyrrol ring lies solely within a largely hydrophobic pocket, containing the residues Ile17, Ile35, Ile43, Ile23, Leu119, Leu14, and Leu37. The closest interactions lie from the MTSL C_6_ to C_D1_ of Ile17, and from the MTSL O to N_D1_ of His120. The addition of MTSL has no significant effect on the backbone structure on the loop around residue 19 relative to PDB 1RY9, nor on the residues with which contacts are made, with the exception of Ile17 (Fig. 2c). In contrast to a number of the reported lysozyme structures, no polar interactions from MTSL S_G_ or S_D_ are apparent with the main chain. A summary of diffraction and refinement statistics is given in Table 1.

The distance between spin-labeled cysteines observable in the crystal structure, taking the measurement from the centremost point of the N–O bond, was 4.5 nm and corresponds exactly to that determined by DEER. This shows that the same conformation of dimer exists in the frozen solution state for the DEER measurements as in the crystalline environment. It provides a further demonstration of the ability of EPR to correctly measure distances between residues of an interacting species.

The crystal structure allowed a comparison of the actual spin-label conformation with those calculated by MMM. The simulations were repeated using the spin-labeled crystal structure (PDB 2XGA) to aid this comparison. In agreement with the original simulations, a DEER distance of 5.3 nm was predicted, and unless stated otherwise, results presented are for these 2XGA simulations rather than those using PDB 1RY9*.* All of the higher-probability conformations placed the label in a less sterically hindered position than the hydrophobic pocket at 175 K. The program did produce a conformation similar to that which was experimentally observed, but only giving it a probability of 0.01 (data not shown).

The top probability conformation at 175 K has a somewhat different χ4 of 91.0 (± 0.1°) to the experimentally determined value of − 51° (± 0.5°) as a result of the stabilization from the MTSL O to the N_D1_ of His120 (angle defined in Fig. 2b). Furthermore, an interaction between MTSL S_D_ and the H on C^α^ seen in a number of lysozyme structures is absent. This allows a greater extension of the label into the hydrophobic pocket for the small price of the loss of this interaction.

MMM simulations are usually performed at the glass transition temperature, as experimental DEER data demonstrate the freezing of conformational distributions below this temperature.^31^ However, repeating the MMM simulations at 50 K and 10 K gave a different result, with the top probability conformation giving a DEER distance of 4.6 nm, close to the experimentally observed value. Despite the similarity in predicted distance, comparison of the MTSL conformation with the crystallographic one showed that they were still dissimilar, meaning that the agreement in DEER distance was due to chance rather than by correct prediction of the MTSL conformation (Fig. 2d).

A comparison between the top probability rotamers calculated at 175 K by MMM using the current crystal structure (PDB 2XGA) and PDB 1RY9 allowed insight into the effect of small side-chain reorientations on the calculations. When using 1RY9 as the basis model, the top probability spin label rotamer (*P* = 0.16) was similar to that of the 2XGA-derived MMM rotamers, pointing away from the hydrophobic pocket and predicting a similar incorrect DEER distance of 5.4 nm. There is a small perturbation in conformation of the label, however, towards Ile17 when the 1RY9 model is used (data not shown). This is a residue that encroaches on the pocket more in 1RY9 than in 2XGA, as can be seen in Fig. 2c. This is a hydrophobic factor possibly weighting this MMM change, but emphasis must be drawn to the fact that this label conformation is also not observed in the 2XGA crystal structure.

This experiment has successfully demonstrated the use of EPR data in studies of protein interactions. Previous conformational work on MTSL has largely focussed on labels within α-helices and the resulting interactions. These are obviously rigid and thus observable in a crystal structure. Trends have therefore been identified pertaining to helix environments, noting particularly such interactions as that with the *i* + 4 residue.^24^ The spin-labeled Spa15 structure demonstrates a very different type of MTSL conformation, dictated not by residues close in sequence, but rather those close in space. The DEER distance measurements agree closely with distances calculated from the crystal structure of the spin-labeled protein, demonstrating it as a useful method for the elucidation of structural phenomena.

### Binding of IpgB1 to Spa15

**Fig. 3:**
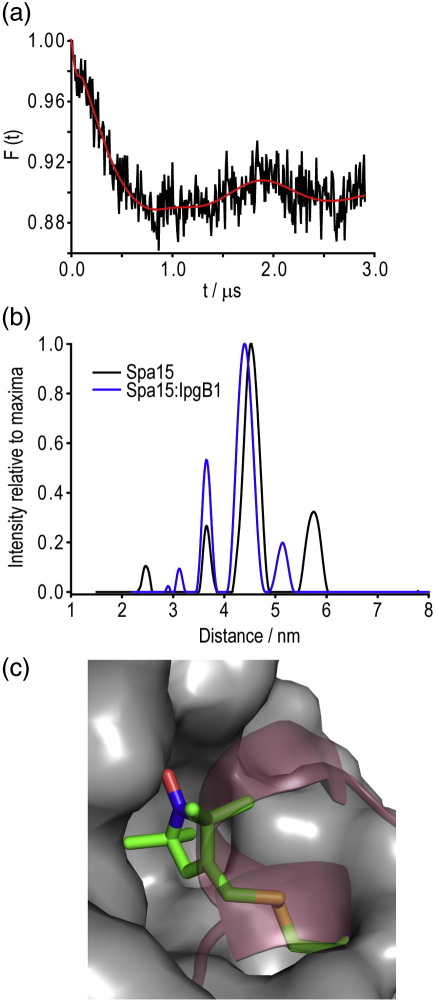
Impact of IpgB1 binding on Spa15 DEER. (a) DEER trace for Spa15:IpgB1. Note: Modulation depths between Fig. 1a and panel (a) of this figure are not directly comparable due to different technical conditions when the samples were measured*.* (b) DEER-derived distances for Spa15 dimer (black) compared to Spa15:IpgB1 (blue) show no significant difference in distance between the Cys19 residues of Spa15 when the effector is bound or unbound. (c) Part of the InvB:SipA structure (PDB 2FM8) with spin-labeled Spa15 (grey surface) replacing InvB. SipA (red ribbon) occludes the binding pocket from the spin label. MTSL is shown in green (atom coloured stick), clashing with SipA.

We were interested in whether DEER could detect a structural change upon binding of the effector IpgB1 to Spa15. The co-purification of Spa15:IpgB1complex at 2:1 ratio was confirmed using multi-angle laser light scattering (data not shown), showing that IpgB1 has no effect on the stoichiometry of the dimeric Spa15. The complex was spin labeled; mass spectrometry confirmed the spin labeling of Spa15 Cys19 in this complex, but not IpgB1 (Cys49 and Cys75) (data not shown). This inability of IpgB1's two cysteine residues to be spin labeled demonstrates their surface inaccessibility in this complex. Spin labeling of the Spa15:IpgB1 complex had no significant effect (± 0.1 nm) on the major DEER distance relative to that of the Spa15 dimer (Fig. 3a and b). Satellite peaks were again observable due to the high concentration conditions.

The complex underwent extensive crystallography trials in order to yield a crystal allowing structural examination around the Spa15 Cys19 residues, but no crystals grew. The absence of any crystals or other Spa15:effector structures required us to look at a homologous protein complex to explain the DEER distance. Using the *Salmonella* sp. Spa15 homologue InvB (33% id.) bound to the chaperone binding domain of the effector SipA allowed us to examine how the effector is expected to bind around the chaperone. In this example, SipA wraps around and uses leucine residues to bind the hydrophobic pocket of InvB, thus presumably occluding the site from a spin-labeled cysteine residue that is conserved at position 19 (Fig. 3c). This is clearly not the case with Spa15:IpgB1, as seen by 100% labeling and a DEER distance unchanged by complexation. Furthermore, from these DEER data, we have shown that Spa15 dimer does not seem to be “compressed” or changed in global conformation upon IpgB1 binding, as a result of the invariant inter-dimer distance.

### Conclusion

To conclude, we show here the invariance of the distance between Spa15 spin-labeled Cys19 residues when uncomplexed and when bound to IpgB1. The effector binding has no constraining effect when it wraps around the chaperone and does not alter the spin-label conformation, demonstrating that Spa15 and IpgB1 cannot interact in the same way as the complex of the InvB/SipA homologue. The spin-label conformation itself has been verified by DEER and crystallography and shown to remain in one favoured position. This position is not that predicted on the basis of the MMM criteria. However, despite the apparent steric limitations of its position relative to a position pointing away from the protein, DEER and crystallography both show that due to hydrophobic interactions, it is the one that is favoured.

## Materials and Methods

### Expression and purification

The *spa15* gene (residues 1–133) was inserted into pET-28b (Nde1/BamH1 sites) to provide an N-terminal His tag. Spa15 was expressed in *E. coli* B834(DE3) with 30 μg/ml kanamycin. The cells were grown at 310 K until *A*_600 nm_ = 0.6, where protein expression was induced by addition of 1 mM IPTG, and cells harvested by centrifugation after 15 h at 293 K. The cells were resuspended in lysis buffer [20 mM Tris–HCl (pH 7.5), 150 mM NaCl] containing complete ethylenediaminetetraacetic acid-free protease inhibitor cocktail (Roche) and subsequently lysed using an Emulsiflex-C5 homogeniser (GC Technology). After retrieving the soluble fraction by centrifugation, the eluant was loaded onto a Ni–NTA Superflow cartridge (Qiagen) where the protein was eluted by an imidazole gradient (between 0 M and 1 M imidazole). Gel-filtration chromatography using a HiLoad 26/60 Superdex 75 column (GE Biosciences) into 20 mM Tris–HCl (pH 7.5), 150 mM NaCl buffer eluted a single dimeric species, whose purity was checked by SDS-PAGE. Mass spectrometry confirmed the molecular weight of the protein to be 17,149 Da (minus N-terminal methionine).

The *ipgB1* gene (residues 1–208) was amplified from *S. flexneri* (M90T) virulence plasmid and subcloned into pACYC-Duet (Nde1/Xho1 sites). Coexpression of a soluble Spa15:IpgB1 complex was achieved by transformation of this pACYC plasmid and the *spa15* containing pET-28b (above) into B834(DE3) containing 30 μg/ml kanamycin and 34 μg/ml chloramphenicol. Overexpression, lysis, and purification of the complex by exploiting the N-terminal His tag of Spa15 were as described for Spa15. The pure complex was obtained by ion-exchange chromatography, using a gradient of 20 mM Tris–HCl (pH 7.5), 20–1000 mM NaCl on a Mono Q 5/50 GL column (GE Healthcare), which separated a single IpgB1:Spa15 complex peak away from a Spa15 dimer peak. For Spa15, SDS-PAGE and mass spectrometry were used to check the purity and integrity of the sample (data not shown).

To spin label both the Spa15 and Spa15:IpgB1 samples, a hundredfold excess of the spin label *S*-(2,2,5,5-tetramethyl-2,5-dihydro-1*H*-pyrrol-3-yl)methyl methanesulfonothioate (MTSL) dissolved in dimethyl sulfoxide was added to the sample and left overnight. Excess label was washed off via repeated centrifugal concentrator washing with 25 mM Tris–HCl (pH 7.5), 50 mM NaCl using Amicon Ultra Ultracel Centrifugal filters (10- kDa cutoff).

### Crystallography and data collection

Crystallisation trials were performed at 294 K by the sitting drop vapour diffusion method. Initial screening was carried out with the Structure Screen 1+2 (Molecular Dimensions), plating the 3 mg/ml protein at 50:50 ratio drops with mother liquor using an OryxNano robot (Douglas Instruments). After initial crystals, including condition 42 (30% polyethylene glycol 8000, 0.2 M ammonium sulfate) and condition 46 (20% polyethylene glycol 8000, 0.05 M potassium phosphate), optimisation led to disc-like crystals for a homemade condition composed of 100 mM guanidium chloride, 100 mM 2-(*N*-morpholino)ethanesulfonic acid (pH 6), 50 mM ammonium sulphate, 4% ethylene glycol. These were cryoprotected in 15% glycerol and X-ray diffracted at beamline I02 (Diamond Light Source, Oxfordshire, UK).

### Structure solution

The data were processed using CCP4 programs; indexing and integration were done with iMosflm^32^ and scaling with SCALA to a resolution of 2.3 Å.^33^ The structure was solved by molecular replacement, using MOLREP^34^ with input model PDB 1RY9.^15^ Iterative refinement (REFMAC^35^) and model building (Coot^36^) to a resolution of 2.3 Å produced the final structure with *R* and *R*_free_ of 22.4% and 26.4%, respectively. The quality of the model was checked using MolProbity,^37^ with a final MolProbity score of 1.08 (100th percentile).

### DEER measurements

The 50% glycerol flash-frozen sample was subjected to the standard four-pulse DEER sequence [π/2(obs)-t1-π(obs)-t-π(pump)-(t1 + t2-t)-π(obs)-t2-echo] on an X-band spectrometer (Bruker ElexSys E680) using a  3-mm split ring resonator (Bruker EN 4118X-MS3) at 50 K. The observer frequency pulse (32 ns length) was 65 MHz higher than the pump frequency pulse (12 ns length). The resonator was overcoupled and the pump pulse coincided with the centre of the microwave mode of the resonator and the maximum of the nitroxide spectrum. Deuterated buffers and glycerol were used. t1 = 400 ns and t2 = 4 μs. t1 was varied eight times by 56 ns to average out any deuterium ESEEM modulations, and the initial π/2 observation pulse was phase cycled. Analysis was carried out using DEERAnalysis2009.^38^

### Multiscale modeling of macromolecular systems

During separate simulations, both the spin-labeled PDB 2XGA of this study and PDB 1RY9 were uploaded to the multiscale modeling of macromolecular systems 2009 program. For the PDB 2XGA, the spin label was removed and readded using the program's labeling tool button. Fifty different possible conformations for the spin label with their associated probabilities were calculated at the glass transition temperature for water/glycerol of 175 K. DEER distances were calculated based on the most likely conformation of spin label, predicting a distance of 5.3 nm with standard deviation of 0.37 nm and relative width of 7.0%. Boltzmann scaling of the full density functional theory calculation of the label at 175 K was applied to predict conformations at 50 K and 10 K. At 50 K, the DEER prediction was 4.73 nm with standard deviation of 0.22 nm and relative width of 4.7%. At 10 K, the DEER prediction was 4.63 nm with standard deviation of 0.10 nm and relative width of 2.2%. For PDB 1RY9 at 175 K, a DEER distance of 5.4 nm was calculated with standard deviation of 0.54 nm and relative width of 9.9%.

### Accession numbers

Coordinates and structure factors have been deposited in the Protein Data Bank. RSCB PDB: 2XGA.

## Figures and Tables

**Table 1:** Data collection and refinement statistics

*Data collection*	
Space group	*P*2_1_
Molecules in asymmetric unit	2
Unit cell parameters (Å, °)	*a* = 56.14, *b* = 50.44, *c* = 57.77, α = γ = 90.00, β = 117.21
Resolution range (Å)	51.4–2.3
No. of unique reflections	12,955
Solvent content (%)	41
*R*_merge_ (%)	11 (31)
*R*_pim_ (%)	10 (28)
Mean *I*/σ(*I*)	5.2 (3.0)
Completeness (%)	99 (99)
Redundancy	3.5 (3.6)

*Refinement summary*	
Resolution (Å)	22.4 (39)
*R*­factor (%)	26.4 (37)
Free *R*­factor (%)	0.008
R.m.s.d. bond lengths (Å)	1.285
R.ms.d. bond angles (°)	4406
No. of atoms in asymmetric unit	22.4 (39)
Ramachandran plot:	
Preferred (%)	99.2
Allowed (%)	0.8
Outliers (%)	0.0
PDB code	2XGA

$R_{\text{merge}}=\frac{\sum_{hkl}\sum_{j}\left|I_{hkl,j}-\langle I_{hkl}\rangle \right|}{\sum_{hkl}\sum_{j}I_{hkl,j}}R_{\text{p}\text{.i}\text{.m}}=\frac{\sum_{hkl}\sqrt{\frac{1}{n-1}}\sum_{j=1}^{n}\sum_{j}\left|I_{hkl,j}-\langle I_{hkl}\rangle \right|}{\sum_{hkl}\sum_{j}I_{hkl,j}}$ where *I*_*hkl,j*_ is the *j*th observation of reflection *hkl* and 〈*I_hkl,j_*〉 is the mean intensity for all observations of *I*_*hkl*_. *n* is the multiplicity of the reflection *hkl*.
